# Obesity among Health-Care Workers: Which Occupations Are at Higher Risk of Being Obese?

**DOI:** 10.3390/ijerph18084381

**Published:** 2021-04-20

**Authors:** Muhammad Syafiq Kunyahamu, Aziah Daud, Nazirah Jusoh

**Affiliations:** 1 Department of Community Medicine, School of Medical Sciences, Universiti Sains Malaysia Health Campus, Kota Bharu 16150, Kelantan, Malaysia; drmuhammadsyafiqk@student.usm.my; 2 Pahang Health State Department, Jalan IM 4, Bandar Indera Mahkota, Kuantan 25582, Pahang, Malaysia; iraaudadi@gmail.com

**Keywords:** obesity, health-care workers, occupational type, obese

## Abstract

Obesity among health-care workers (HCWs) is an important issue as it can affect both their health condition and their professional capability. Although adult obesity is attributable to occupational factors, few reports are available on Malaysian health-care workers’ obesity and whether different health-care job categories are related to workers’ obesity. This study aimed to determine the prevalence of obesity among HCWs and the association between various HCW job categories and obesity. A cross-sectional study was conducted by analyzing secondary data from the 2019 annual cardiovascular health screening program, which included information regarding all government health-care workers in the east coast region of Peninsular Malaysia. The subject’s body mass index (BMI) was categorized according to WHO criteria. Only 43% of the subjects had a normal BMI, while 33.1% were categorized as overweight, and 21.1% were obese. Different HCWs’ job categories were shown to be significantly associated with their obesity status, with nurses apparently having a higher risk of being obese (Adj OR = 1.91, 95% CI 1.45, 2.53, *p*-value < 0.001). This study’s results require further exploration of HCWs’ working condition factors and for different job categories that contribute to obesity. Public health intervention programs to combat obesity should be implemented that primarily target HCW groups at the highest risk of obesity.

## 1. Introduction

Recently, obesity has become a significant public-health threat with prevalence increasing worldwide. As with other types of profession, HCWs are also affected by obesity. Health-care workers (HCWs) should be role models who increase community awareness of obesity prevention and encourage patients to change their behavior towards a healthy lifestyle. Despite working in an environment related to disease prevention and health promotion, HCWs in several studies have shown a trend towards obesity over time [1,2] and to have higher levels of obesity than the general population [3,4,5,6,7]. 

Different job categories of HCWs bear different risks of becoming obese. Different job categories of HCWs have different work scope areas which will indirectly contribute to a greater risk of obesity among HCWs [8,9,10]. Obesity among doctors may affect patients’ perception of their credibility in advising patients, especially regarding lifestyle modification among overweight or obese patients [11]. Bleich et al. stated, in their study, that obese doctors are less confident in providing healthy nutrition and exercise advice to their patients [12].

In Malaysia, little research has been done on a large scale to study obesity among HCWs. Some studies have reported that HCWs in Malaysia have a higher risk of obesity than the general adult population [6,7,13,14]. However, to the best of our knowledge, existing research has not explored whether specific job categories among Malaysian HCWs are related to their obesity status.

An accurate assessment of this issue has become essential as it will affect health services, directly or indirectly. Knowing the higher-risk occupation groups among HCWs can contribute towards the preparation of intervention programs that address this issue. This study may also help other researchers in studying additional factors contributing to obesity among certain job categories of HCWs. Therefore, this study’s main objective was to determine the prevalence of obesity among HCWs and the potential associations between specific HCW job categories and obesity.

## 2. Materials and Methods 

### 2.1. Study Design and Samples

A cross-sectional study was carried out in the east coast region of Peninsular Malaysia. For this study, analysis was conducted using the secondary data from the annual cardiovascular health screening program for all government HCWs, covering all health clinics and hospitals in eleven districts of the east coast region of Peninsular Malaysia. All permanent government HCWs who underwent a health screening program between 1 January and 31 December 2019 were eligible to be included in this study. Data sets with no information on job category, weight, or height were excluded. 

The included HCWs were divided into three groups: doctors (house officers, medical officers, specialists), nurses, and other job categories (pharmacists, medical assistants, occupational therapists, physiotherapists, health assistants, medical laboratory technologists, dietitians, and nutritionists). Using the body mass index (BMI), HCWs were divided into various BMI categories using the WHO 1998 classifications: underweight (BMI less than 18.5 kg/m^2^), normal (BMI 18.5 to 24.9), overweight (BMI 25 to 29.9), and obese (BMI equal to or greater than 30 kg/m^2^).

The sample size estimation was 5540, as calculated by the independent two-proportion formula of power and sample size calculation software. The research power was fixed at 90% and the confidence interval (CI) at 95%. P0 was the proportion of exposure among the non-obese group of doctors, with 61% [7]. Meanwhile, P1 was set as 55%, with the ratio of the non-obese group to the obese group being four to one. The calculated sample size was established considering an allowance of 20% for possible missing or incomplete data. However, since only 4489 study subjects were available, all samples were included in the study analysis, and no sampling method was applied.

### 2.2. Ethical Approval and Consent

Ethical approval was granted by the Human Research and Ethics Committee (HREC), Universiti Sains Malaysia (USM/JEPEM/19100596), and the Medical Review and Ethical Committee of the National Institute of Health, Ministry of Health Malaysia NMRR-19-3225-51278 (IIR). The local state health director permitted the use of the data from the annual cardiovascular health screening program in this study.

### 2.3. Statistical Analysis

Data were uploaded from Microsoft Excel, and the analysis was performed using SPSS Version 24.0. Any missing values or errors that needed correction were detected using a preliminary data description. Based on their normal distribution, numerical data were presented as mean (SD), and categorical data were presented as frequency (percentage). Descriptive statistics for all variables were calculated for each group. HCWs with BMIs greater than or equal to 30 kg/m^2^ were classified as the obese group, and those with BMIs of less than 30 kg/m^2^ were classified as the non-obese group.

All significant variables (*p* < 0.25) from the univariable analysis were selected for multiple logistic regression. A stepwise backward elimination method was used to identify the final logistic regression model of the association between job category and obesity among HCWs. The final model was tested for two-way interaction and multicollinearity. A *p*-value < 0.05 was established as statistically significant. 

## 3. Results

### 3.1. Sociodemographic Data 

After applying the inclusion and exclusion criteria, only 4241 (94.47%) samples were extracted from the data. Hence, all 4241 samples were included in the analysis. The study subjects’ mean age was 35.64 years, and most subjects were Malays (93.5%). Most (75.3%) were female HCWs, and 2680 (63%) worked in hospitals. Furthermore, 7.5% of the subjects had comorbidities, such as diabetes mellitus, asthma, hypertension, and cardiovascular heart disease. Additionally, only 4.7% of the study populations were smokers. In terms of job category distribution in this study, only 12.3% of the subjects were doctors; 45.4% were nurses, and 42.3% were in other job categories (Table 1). 

### 3.2. Prevalence of Obesity among HCWs

Based on the WHO BMI classification, 1405 (33.1%) of the subjects were overweight, and 892 (21.1%) were obese. The mean BMI was 26.14. The prevalence of obesity among HCWs in this study was 21.1%. Among the obese subjects, 73 (1.7%) had a BMI of over 40.0, which is categorized as morbidly obese (Table 2). In comparing the three job categories, it was found that the prevalence of obesity was higher among nurses (50%) than doctors (7.6%) and other job categories (42.4%) (Table 3).

### 3.3. Association of Job Categories and Obesity among HCWs

The results for univariable analysis were summarized in Table 4. Multivariate analyses using multiple logistic regression confirmed a significant association between the job categories of HCWs and obesity. From this analysis, we can conclude that only variable job categories and comorbidity were significantly associated with obesity among HCWs. In terms of association with job category, the statistically significant results remained the same after adjusting for comorbidity and after other variables were controlled. In other words, from this model, we can interpret that after other variables were controlled, nurses had a 1.91 times odd (95% CI 1.45, 2.53) of becoming obese compared to doctors, when adjusted for comorbidity. Other job categories had a 1.63 times odd (95% CI 1.23, 21.6) of becoming obese compared to doctors, when adjusted for comorbidity (Table 5). 

## 4. Discussion

### 4.1. Characteristics of the Study Population

Overall, this study’s results can be used to predict the long-term effect of obesity on non-communicable disease status among HCWs in the future; the average age of the study population was 35.64 years which suggests that most HCWs in this study will have an average of at least 25 more years of mandatory service with the Ministry of Health Malaysia.

Our findings of unequal HCW gender distribution is consistent with other studies [5,6,7,8,15]. In terms of the unequal distribution of job categories found in our study, it was also reported in another Malaysian local study that 73.4% of HCWs came from nursing categories [6]. This may be attributed to the larger number of nurses employed in the Malaysian health-care sector as compared to other HCW professions [16]. Therefore, the high ratio of females to males and the predominance of the nurse job category found in this study, represents the actual gender and job category distribution among HCWs in Malaysia.

As we all know, females made up the majority of the field of nursing [17]. Even though our study did discover that female HCWs constituted up to 75.3% of the participants and that nurses had the highest risk of developing obesity, we were unable to prove any significant association between HCWs’ gender and obesity. The results of this study were similar to a study conducted by Hales et al. [18]. However, our results were different to most of the other studies that discovered that women are at greater risk of being obese [3,15,19,20]. Our findings might be affected by the disproportionate distribution of male and female HCWs in our study, even though it represents the actual gender distribution of HCWs in Malaysia.

### 4.2. Prevalence of Obesity among HCWs 

Obesity among HCWs is an important issue as it impacts the morbidity of HCWs. This study showed that one in five HCWs were obese, which is not much different from other Malaysian local studies done among HCWs [6,7]. These findings revealed that, despite the many initiatives undertaken by the Malaysian Ministry of Health, the prevalence of obesity among HCWs in Malaysia is not declining.

Compared to other countries in Southeast Asia, there is a marked disparity in the prevalence of obesity. Studies in Thailand and Singapore showed a much lower burden of obesity among HCWs, with a prevalence of 6.5% [21] and 6.3% [22], respectively. This could be because the general population of Malaysia has one of the highest prevalence rates of obesity of the Asian countries [23,24]. Furthermore, the prevalence of obesity among the adult populations of Thailand and Singapore is very low compared to that of Malaysia [23,25,26]. 

Overall, the prevalence of obesity among HCWs was higher than the latest national obesity rates for the general Malaysian adult population, recorded at 19.7% [27]. This is consistent with other international studies done by Iwuala et al. [4] and Sharma et al. [5], in which the HCWs’ obesity prevalence rates were higher than those of the general adult population in their respective regions. Another local study, by Hazmi et al. [6], also showed a higher burden of obesity among HCWs than the national prevalence reported in the same year.

The higher burden of obesity among HCWs compared to the general adult population could be due to a greater vulnerability to obesity because of their exposure to irregular and prolonged working hours, poor diet, and stress at work [9,28]. A study by Luckhaupt et al. [2] also discovered that among all industry categories in the United States, people that work in the health-care industry are associated with a significantly higher risk of obesity.

### 4.3. Association with Job Categories

Similar to our study’s results, a higher risk of obesity for nurses compared to other job categories, has been reported in other studies. In their studies in England and Scotland, Kyle et al. [9,10] discovered that the risk of obesity was lower for other HCW job categories than for nurses. Unfortunately, Hegde et.al in their studies in Tamil Nadu, India, found results that contradicted with our study, showing that the burden of obesity was more significant among doctors compared to nurses [8]. Overall, all the studies mentioned above agreed that different job categories had an association with obesity among HCWs. 

However, the above studies did not share comparable sociodemographic populations to that of Malaysia. Most local studies among HCWs did not specifically analyse different types of HCW occupations and obesity; Hazmi et al. [6] only mentioned the overall prevalence of obesity without further analysis according to job category, while Ghazi et al. [13] and Ramli et al. [14] only divided the occupations of HCWs into professional versus ancillary jobs. In contrast, our study further classified HCW occupations into three main groups—doctors, nurses, and other job categories—thus, we know that nurses specifically are at greater risk of becoming obese, compared to doctors and other job categories in Malaysia.

This higher risk of obesity for nurses may be due to factors related to their job conditions; nurses must do shift work with disruptive working patterns to deliver health-care services. Many studies have discovered that obesity is one of the adverse health effects of shift work [28,29,30,31,32]. In their study, Liu et al. [30] reported that the risk of being obese increased by 17% for workers doing shift work compared to those doing non-shift work. Shift work that disrupts sleep patterns, dietary habits [33], and exercise may make it more challenging for HCWs to stay fit and healthy. Generally, shift-working nurses are less likely to have regular leisure-time physical activity [34]. Owing to their irregular work schedules, maintaining an exercise regime outside of work is impractical. A study in Poland found that nurses who worked more than eight night shifts per month were at greater risk of developing obesity [29]. 

Unfortunately, due to the limited availability of secondary data, we were unable to perform a comparison of obesity risk between shift and non-shift HCWs by job category. We were also unable to explain the finding from our study that HCWs’ workplace was not significantly related statistically to obesity, even though more HCWs, particularly nurses, who work in hospitals must do more shift work than HCWs working in primary health clinics, who work office hours. This could have affected our data on HCWs working in hospitals, including those in professions that do not require shift work, such as doctors. In Malaysia, doctors in both hospitals and primary health clinics usually work office hours and only occasionally work overnight on call. Therefore, further research is required to compare nurses’ working environments and working hours with those of other occupational groups who work shifts, in order to get a better understanding of how these factors affect their obesity risk.

## 5. Conclusions

The prevalence of obesity among HCWs in the east coast region of Peninsular Malaysia was higher than in the general adult population. The prevalence of obesity was significantly higher in nurses as compared to doctors and other job categories. HCWs’ job categories were found to be significantly associated with obesity, and different HCWs seem to have different risks; being a nurse significantly increases the risk of being obese compared to other HCW job categories. Therefore, more effective interventions and policy implementations are required for the different types of HCWs because being obese carries a significant risk for non-communicable diseases and will ultimately affect health-care services. 

## Figures and Tables

**Table 1:** Sociodemographic characteristics of study subjects (*n* = 4241).

Variables	Mean (SD)	*n* (%)
Age (years)	35.64 (7.37)	
Gender		
Male		1048 (24.7)
Female		3195 (75.3)
Ethnicity		
Malay		3964 (93.5)
Non-Malay ^a^		277 (6.5)
Marital Status		
Married		3655 (86.2)
Unmarried ^b^		586 (13.8)
Workplace		
Hospital		2680 (63.2)
Non-Hospital ^c^		1561(36.8)
Occupation		
Doctors ^d^		521 (12.3)
Nurses		1924 (45.4)
Others ^e^		1796 (42.3)
Comorbid ^f^		
No		3925 (92.5)
Yes		316 (7.5)
Smoking status		
Non-smoker		4042 (95.3)
Smoker		199 (4.7)

^a^ Chinese, Indian and others; ^b^ Divorced, single, widowed; ^c^ Primary health clinic, community clinic; ^d^ House officer, medical officer, specialist; ^e^ Pharmacist, occupational therapist, medical assistant, physiotherapist, health assistant, medical laboratory technologist, dietitian, nutritionist; ^f^ Diabetes mellitus, hypertension, heart disease, asthma.

**Table 2:** BMI status among health-care workers (*n* = 4241).

Variable	BMI (kg/m^2^)	Mean (SD)	*n* (%)
BMI Status		26.14 (5.09)	
Underweight	<18.5		119 (2.8)
Normal	18.5–24.9		1825 (43.0)
Overweight	25.0–29.9		1405 (33.1)
Obese	≥30		892 (21.1)
Obesity Class I	30.0–34.9		647 (15.3)
Obesity Class II	35.0–39.9		172 (4.1)
Obesity Class III	≥40		73 (1.7)

BMI: Body mass index.

**Table 3:** Obesity by job categories (*n* = 4241).

	Obese (*n* = 892)	Non-Obese (*n* = 3349)	*p*-Value
Variables	*n* (%)	*n* (%)	
Job Category			
Doctors ^a^	68 (7.6)	453 (13.5)	
Nurses	446 (50.0)	1478 (44.1)	
Others ^b^	378 (42.4)	1418 (42.3)	<0.001 ^c^

^a^ House officer, medical officer, specialist; ^b^ Pharmacist, occupational therapist, medical assistant, physiotherapist, health assistant, medical laboratory technologist, dietitian, nutritionist; ^c^ Chi-square.

**Table 4:** Simple logistic regression of the association between each variable and HCWs’ obesity status (*n* = 4241).

Variables	Regression Coefficient B	Crude OR (95% CI)	Wald Statistics (df)	*p*-Values
Age	0.047	1.05 (1.04, 1.06)	90.81 (1)	<0.001
Gender				
Male		1		
Female	−0.072	0.93 (0.79, 1.10)	0.70 (1)	0.40
Ethnicity				
Malay		1		
Non-Malay ^a^	−0.423	0.66 (0.47,0.92)	6.07 (1)	0.014
Marital Status				
Married		1		
Unmarried ^b^	−0.438	0.65 (0.51, 0.82)	13.01 (1)	<0.001
Workplace				
Non-Hospital		1		
Hospital	0.17	1.18 (1.02, 1.38)	4.67 (1)	0.03
Smoking status				
No		1		
Yes	0.215	1.24 (0.89, 1.73)	1.62 (1)	0.204
Comorbid ^c^				
No		1		
Yes	1.254	3.51 (2.78, 4.44)	109.06 (1)	<0.001
Job Category				
Doctors		1		
Nurses	0.698	2.01 (1.53, 2.65)	24.58 (1)	<0.001
Others	0.574	1.77 (1.34, 2.35)	16.27 (1)	<0.001

^a^ Chinese, Indian and others; ^b^ Divorced, single, widowed; ^c^ Diabetes mellitus, hypertension, heart disease, asthma.

**Table 5:** Multiple logistic regression modelling of factors associated with obesity among HCWs.

Variables	Regression Coefficient B	Adjusted OR (95% CI)	Wald Statistics (df)	*p*-Values
Job Category				
Doctors ^a^		1		
Nurses	0.649	1.91 (1.45, 2.53)	20.92 (1)	<0.001
Others ^b^	0.488	1.63 (1.23, 2.16)	11.53 (1)	0.001
Comorbid ^c^				
No		1		
Yes	1.234	3.43 (2.71, 4.35)	104.35 (1)	<0.001

^a^ House officer, medical officer, specialist. ^b^ Pharmacists, occupational therapist, medical assistant, physiotherapist, health assistant, medical laboratory technologist, dietitian, nutritionist; ^c^ Diabetes mellitus, hypertension, heart disease, asthma. Constant = −1.956. The enter method was applied to identify the final model. No multicollinearity and no interaction were noted between the variables. Hosmer–Lemeshow test: *p*-value = 0.722. Classification table, 79.0% correctly classified. The area under the Receiver Operating Characteristics (ROC) curve was 58.7%.

## Data Availability

Data Sharing is not applicable to this article.
